# Impact of the 2009 US Preventive Services Task Force Guidelines on Screening Mammography Rates on Women in Their 40s

**DOI:** 10.1371/journal.pone.0091399

**Published:** 2014-03-11

**Authors:** Amy T. Wang, Jiaquan Fan, Holly K. Van Houten, Jon C. Tilburt, Natasha K. Stout, Victor M. Montori, Nilay D. Shah

**Affiliations:** 1 Division of General Internal Medicine, Mayo Clinic, Rochester, Minnesota, United States of America; 2 Knowledge and Evaluation Research Unit, Mayo Clinic, Rochester, Minnesota, United States of America; 3 Division of Health Care Policy & Research, Mayo Clinic, Rochester, Minnesota, United States of America; 4 Department of Population Medicine, Harvard Medical School and Harvard Pilgrim Health Care Institute, Boston, Massachusetts, United States of America; 5 Division of Endocrinology, Mayo Clinic, Rochester, Minnesota, United States of America; University of Pennsylvania, United States of America

## Abstract

**Background:**

The 2009 US Preventive Services Task Force breast cancer screening update recommended against routine screening mammography for women aged 40–49; confusion and release of conflicting guidelines followed. We examined the impact of the USPSTF update on population-level screening mammography rates in women ages 40–49.

**Methods and Findings:**

We conducted a retrospective, interrupted time-series analysis using a nationally representative, privately-insured population from 1/1/2006-12/31/2011. Women ages 40–64 enrolled for ≥1 month were included. The primary outcome was receipt of screening mammography, identified using administrative claims-based algorithms. Time-series regression models were estimated to determine the effect of the guideline change on screening mammography rates. 5.5 million women ages 40–64 were included. A 1.8 per 1,000 women (p = 0.003) decrease in monthly screening mammography rates for 40–49 year-old women was observed two months following the guideline change; no initial effect was seen for 50–64 year-old women. However, two years following the guideline change, a slight increase in screening mammography rates above expected was observed in both age groups.

**Conclusions:**

We detected a modest initial drop in screening mammography rates in women ages 40–49 immediately after the 2009 USPSTF guideline followed by an increase in screening rates. Unfavorable public reactions and release of conflicting statements may have tempered the initial impact. Renewal of the screening debate may have brought mammography to the forefront of women's minds, contributing to the observed increase in mammography rates two years after the guideline change. This pattern is unlikely to reflect informed choice and underscores the need for improved translation of evidence-based care and guidelines into practice.

## Introduction

In November 2009, the United States Preventive Services Task Force (USPSTF) issued updated screening mammography guidelines which recommended “against routine screening mammography in women aged 40 to 49 years (C recommendation) [1],” marking a major change from their previous recommendation of routine screening mammography every 1 to 2 years starting at age 40. In December 2009, this wording was revised to “the decision to start regular, biennial screening mammography before the age of 50 years should be an individual one and take patient context into account, including the patient's values regarding specific benefits and harms [1].” This guideline shift triggered an intense national discussion with vociferous dissent from the public as well as physicians, and prompted many professional organizations including the American Cancer Society to reaffirm their differing positions on screening mammography [2].

Given the renewed debate around the benefits and harms of mammography in women in their 40s and the wide spectrum of responses and reactions to the guidelines both among the public, health care professionals and organizations, we aimed to determine to what extent the updated USPSTF guidelines affected the utilization of screening mammography among women in their 40s.

## Methods

### Ethics statement

This study was deemed exempt by Mayo Clinic Institutional Review Board. Data are from the IMS LifeLink Health Plan Claims Database provided by IMS Health (www.imshealth.com) and is hosted at the Mayo Clinic, Rochester, MN. The data was de-identified by IMS before it was made accessible to Mayo Clinic. The data are provided to the Mayo Clinic through a data use agreement from IMS Health and thus we cannot share data. Researchers would be able to obtain data directly from IMS Health through a data use agreement and licensing fees.

### Study sample

We conducted an interrupted time-series analysis utilizing administrative data from the IMS LifeLink Health Plan Claims Database (formerly PharMetrics; Danbury, CT) to evaluate the impact of the USPSTF guidelines on screening mammography rates. This longitudinal, patient-level database is one of the largest integrated claims databases for commercial insurance in the US and contains medical and pharmaceutical claims data for more than 80 million members from over 100 health plans, including both traditional and managed care plans [3]. It has been shown to be nationally representative of the commercially insured US population in various demographic measures including geographic region [4]. The database provides basic demographic information, administrative information related to the receipt of medical care including medical diagnoses using ICD-9 (International Classification of Diseases, 9th revision) coding system, and procedure codes, using the CPT-4 (Current Procedural Terminology-4) system.

The study sample included women aged 40 to 64 years old with at least 1-month of enrollment in the IMS LifeLink database from January 1, 2006 to December 31, 2011. Women 65 and older were excluded from the study because the IMS LifeLink database does not fully capture Medicare data. The start date of January 2006 was chosen in order to account for possible effects of the recent economic recession and because it offers almost four years of data prior to the USPSTF breast screening recommendations to establish an adequate baseline and secular trend. The end date of December 2011 was chosen in order to detect not only the short-term impact but also the longer-term effect of the USPSTF update. The six-year timeframe also allowed for proper evaluation of seasonal fluctuations for mammography utilization. To strengthen the comparison, we examined rates of screening pap smears and tetanus immunizations to account for trends in preventive behavior as a control group.

### Identification of screening mammograms

**Figure 1:**
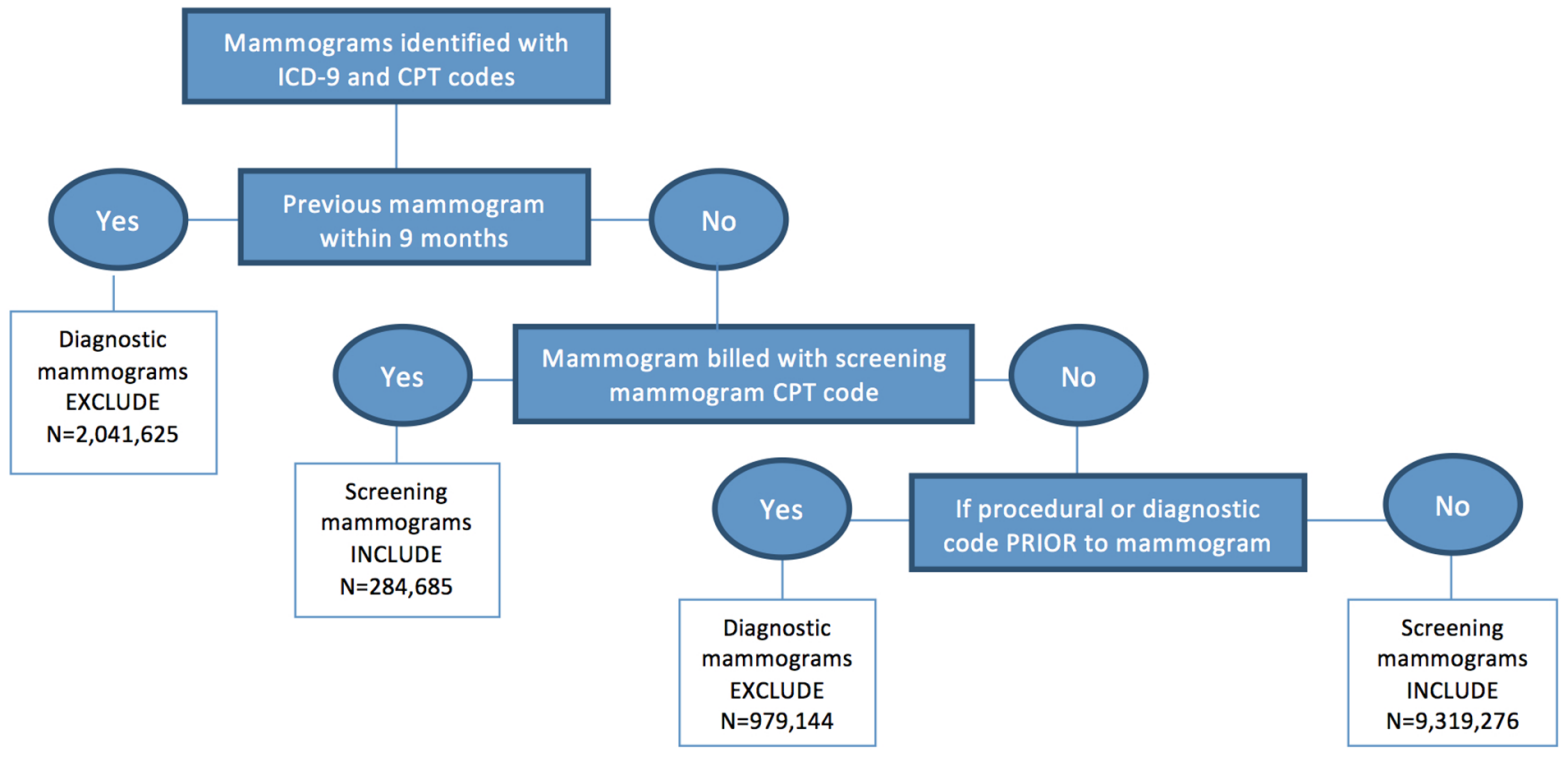
Screening algorithm used to identify screening mammograms. ICD-9, International Classification of Diseases, 9th revision; CPT, Current Procedural Terminology.

Previous studies have developed and validated algorithms for identifying screening mammograms using claims data [5], [6]. Based on these studies, we developed claims-based algorithms to determine the number of screening mammograms per month (Figure 1). Codes for both screening and diagnostic mammograms were included in the algorithm as coding may not be reliable for differentiating between screening and diagnostic mammograms [7]. We also identified the number of enrollees for each month from January 2006 to December 2011. Mammograms were identified using HEDIS (Healthcare Effectiveness Data and Information Set) and included CPT codes (76090, 76091, 76092, 77055, 77056, 77057) and ICD-9 diagnosis codes for screening mammography (V76.11, V76.12). If there were duplicate claims, more than one mammogram billed for the same woman on the same day, this was counted as one mammogram. Our outcome measure was defined as the monthly screening mammography rate per 1,000 women. Mammography rates were calculated on a monthly basis by dividing the number of screening mammograms performed in a particular month divided by the total number of eligible women in that month. This rate was calculated overall and then stratified by age group (40–49 years, 50–64 years). Age was calculated based on the service year, thus women moved from one age group to the other the year they turned 50.

### Statistical analysis

We used an interrupted time-series approach with segmented linear regression models [8] to estimate the change in trends of monthly screening mammography rates per 1,000 women for age group 40–49 and 50–64 separately (Text S1. Statistical Appendix). We defined two time segments: a baseline period (January 2006 to October 2009) and a post-USPSTF update period (November 2009 to December 2011). The models include a constant term to estimate the mammography rate at baseline, a linear time trend, and an indicator for the post-USPSTF update period (0:before update, 1:after update). The impact of the USPSTF update is estimated by the regression coefficient of the indicator variable, which represents a level shift in the rate of mammography screening at the time of the USPSTF update. Mammography screening rates at two years after the update were estimated by fitting the time series models to the observed monthly rates of mammography screening. These observed rates were compared to expected rates. Expected rates were calculated by using the baseline trend prior to the guideline change to predict rated through December 2011. Strong seasonal fluctuations were adjusted by including an autoregressive error of the order of 12 months. Models were fit using SAS PROC AUTOREG using SAS version 9.2 (SAS Institute Inc., Cary, NC). Given that the intervention of interest was a guideline change which may have a lagged effect, compared to a policy change, which may have a more immediate effect, we used a 2-month post guideline period to assess for the immediate impact of the guideline. Sensitivity analyses with alternate number and cut-off dates of time periods and accounting for effects of the economic recession were also planned (Text S2. Sensitivity Analyses).

## Results

The IMS LifeLink database included 11.4 million unique women during this timeframe. Of these, 5,514,038 women met eligibility criteria, 2,177,343 women in the 40–49 age group and 3,336,695 women in the 50–64 age group. The average baseline monthly screening mammography rate from January 1, 2006 to October 31, 2009 was 34.2 per 1,000 enrolled women per month in the 40–49 age group and 42.9 per 1,000 enrolled women per month in the age 50–64 age group.

### Screening mammography rates in the 40 to 49 age group

**Figure 2:**
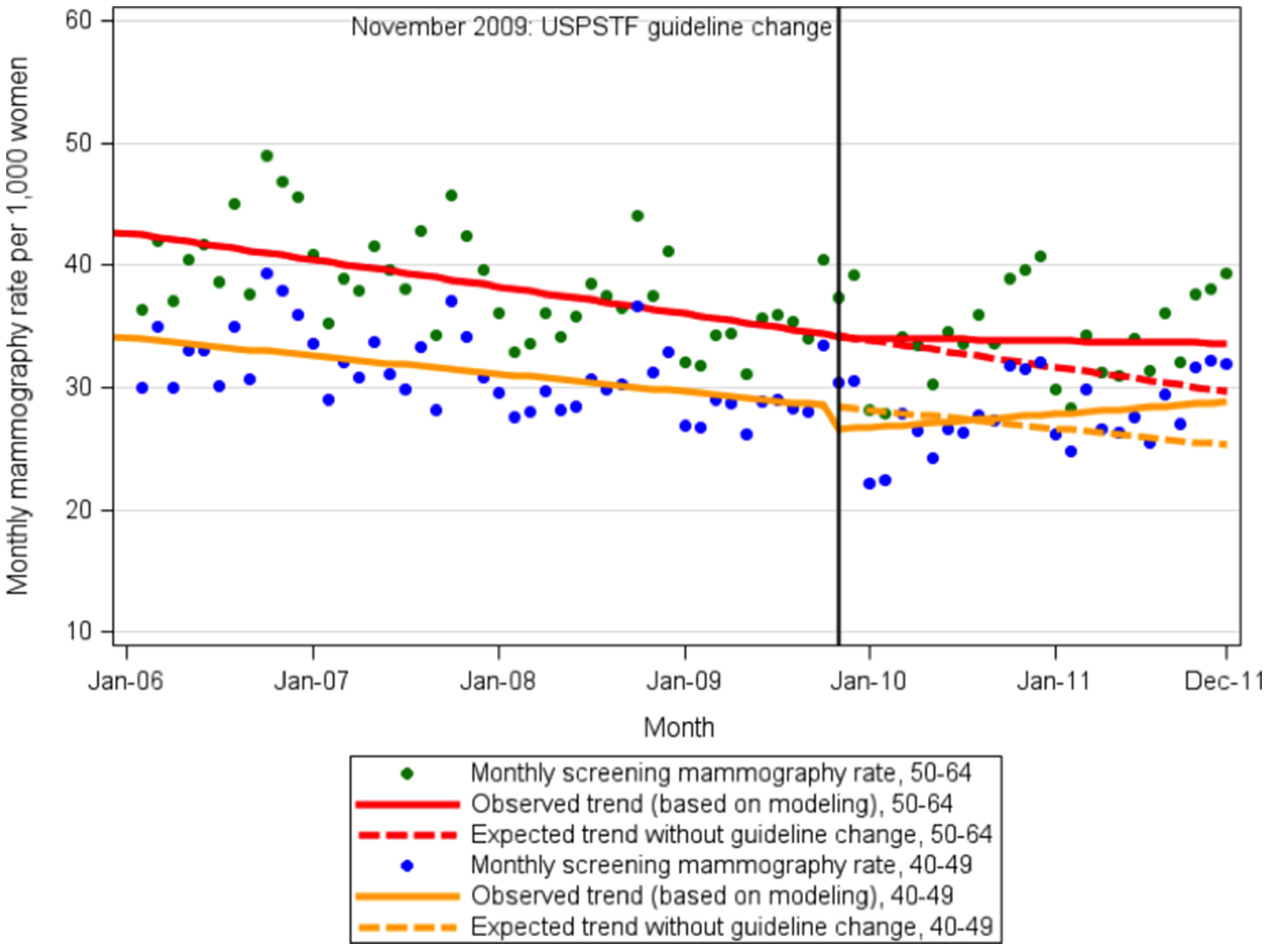
Screening mammography rates before and after the US Preventive Services Task Force guideline change. The solid lines represent the observed screening mammography rates in women ages 40–49 and 50–64 before and after the change in breast cancer screening guidelines by the US Preventive Services Task Force in November 2009. The dotted lines represent the modeled expected screening mammography rates in the respective age groups after the guideline change.USPSTF, United States Preventive Services Task Force.

Based on the projected trend post-guideline change, we found an initial 1.8 per 1,000 (p = 0.003) enrolled women per month decrease in screening mammography rates in the 40–49 age group two months after the update (Table 1, Figure 2). During the two-year period following the guideline update, the screening mammography rate in the 40–49 age group increased at a rate of 0.09 per 1000 women per month (p<0.017). By December 2011, the observed screening mammography rate was 31.9 per 1000 women per month compared to the predicted rate of 25.4 per 1000 women per month based on the screening mammography trend prior to the update. Changing the defined cut-off dates for the post-guideline change period did not alter our results (TextS2. Sensitivity Analyses). In comparison, there was no significant change in Pap smear rates during the initial two-month period (Figure S1). Tetanus rates were also unchanged in this age group.

**Table 1:** Screening mammography utilization rates by age 40–49, 2006–2011.

Women ages 40–49	Estimate	Standard Error	95% CI	p-value	
Baseline mammography screening rate (intercept)	34.24/1000 women	0.91	(32.46, 36.02)		<0.001
Change in screening rate in the 2-months after guideline release	−1.81/1000 women	0.59	(−2.96, −0.65)		0.003
Trend in monthly screening rate prior to the guideline release	−0.12	0.02	(−0.17, −0.08)		<0.001
Trend in monthly screening rate 2-years after the guideline release	0.09	0.04	(0.02, 0.16)		0.017

CI: Confidence interval.

### Screening mammography rates in the 50 to 64 age group

In contrast, there was no significant change detected in the screening mammography rate in the 50–64 age group in the immediate two-month period after the guideline change (Table 2, Figure 2). By December 2011, the observed mammography screening rate was 39.3 per 1000 women per month compared to the predicted rate of 29.7 per 1000 women per month based on the screening mammography trend prior to the update. There was no significant change in pap smear rates during the initial two-month period (Figure S1). Tetanus rates remained unchanged among women 50–64.

**Table 2:** Screening mammography utilization rates by age 50–64, 2006–2011.

Women ages 50–64	Estimate	Standard Error	95% CI	p-value	
Baseline mammography screening rate (intercept)	42.86/1000 women	1.22	(40.46, 45.25)		<0.001
Change in screening rate in the 2-months after guideline release	−0.13/1000 women	0.68	(−1.47,1.20)		0.85
Trend in monthly screening rate prior to the guideline release	−0.18	0.03	(−0.24, −0.13)		<0.001
Trend in monthly screening rate 2-years after the guideline release	−0.018	0.04	(−0.10,0.07)	0.67	

CI: Confidence interval.

## Discussion

This is the first study to quantify the potential effects of the 2009 USPSTF breast cancer screening recommendations on screening mammography among privately insured women. The recommendations changed from routine screening mammography every 1–2 years for women 40 and over to routine screening mammography for women 50 and over and engaging in individualized decision making for women ages 40–49 [1]. These new recommendations are more aligned with international screening practices, but differ from guidelines by other organizations in the US. The initial impact on screening rates is consistent with the context of the update. We detected a modest initial drop in screening mammograms for women in the 40–49 age group, consistent with a more substantial recommendation change for this age group, compared to no impact on mammography rates in the 50–64 age group, reflecting the more subtle change from annual to biennial screening mammography.

Two previous studies have estimated effects of the guideline change using self-report data and found that the guidelines had no effect on screening mammography rates [9], [10]. However, self-reported survey data is reported to be strongly upwardly biased compared to administrative data [11].

Our results also allay concerns that the major recommendation shift in the 40–49 age group would have a ripple effect, decreasing screening mammography among women over 50. In fact, we observed the converse; the guideline change was associated with an increase in screening mammography rates during the two-year period after the update in all age groups. This may be attributable to the renewal of the screening mammography debate in women ages 40–49, which has resulted in more intense and frequent media coverage of mammography since the guidelines were released. The increase in news coverage may have made screening mammography more prominent in women's minds and may have served as a persistent reminder to get a mammogram.

Public resistance to the update, possibly fueled by negative portrayal of the USPSTF guidelines in the media [12] and the subsequent release of numerous conflicting guidelines, may have hindered the translation of these recommendations into practice. The American Cancer Society (ACS) immediately reaffirmed its previous guidelines when the USPSTF update was released [2], continuing to recommend routine annual screening mammography for women ages 40–49 as did the National Comprehensive Cancer Network (NCCN) [13]. The American College of Surgeons officially supported the ACS guidelines in January 2010 [14]. The American College of Radiology and Society for Breast Imaging released a joint statement in January 2010 [15] and in August 2011, the American College of Obstetricians and Gynecologists (ACOG) released guidelines both in agreement with the ACS guidelines [16]. The American College of Physicians 2007 guidelines already reflected the sentiments of the updated USPSTF recommendations [17].” Two organizations released guidelines in alignment with USPSTF: American Academy of Family Physicians in January 2010 and Kaiser Permanente Care Management Institute in August 2010 [18]. Perhaps, the issuance of multiple guidelines also had a similar effect as the constant media coverage, placing screening mammography at the forefront of patients and clinicians' minds.

We found decreasing screening mammography rates from 2007 to early 2009 which coincide with the timing of the recent Great Recession. Dorn et al has shown that the recent economic recession had a negative impact on colonoscopy screening rates [19]. Screening mammography rates dipped beyond expected with the effects of the recession in the months following the guideline update for women ages 40–49 (November 2009) but then began to increase for both age groups, surpassing expected rates, which is likely in part attributable to the economic recovery. The overall trends in screening mammography rates coincide nicely with both the downward and upward trajectories of the economic recession and recovery.

Women in their upper-40s may have become accustomed to annual screening mammography prior to the update and could be more resistant to adjusting screening practices than women turning 40. To explore this further, we analyzed yearly screening mammography rates by within smaller age groups, 40–42, 43–45, and 46–49. We found that the screening patterns were similar for 43–45 and 46–49 year-old women, with a small drop after the guidelines and then decreasing at a slower rate in the two-year period following the guidelines. On the other hand, the 40–42 age group showed a steeper decline immediately after the guidelines and then increased over the subsequent two years. It is possible that these younger women initially held off after the USPSTF guidelines were released then changed their minds possibly due to increased mammography coverage, conflicting guidelines, the economic recovery or other factors.

For comparison purposes, we also looked at rates of other screening tests and preventive care for this age group. We did not detect any drop in cervical cancer screening rates after the USPSTF breast cancer screening update, but we also saw a slight increase in Pap rates over the following two years after the update. This slight increase may be fueled by the same factors resulting in increased screening mammography rates, including media coverage, economic recovery, and perhaps women getting Pap smears when going in for mammograms. Cervical cancer screening guidelines also underwent a change during this same time period; ACOG updated guidelines online in November 2009 and in print in December 2009. However, the major change in this update was directed at women under 30 and over age 65 [20]. For women aged 30–64, the recommendation was updated from every 2–3 year pap smears to every 3 years, which is unlikely to affect our study population. Tetanus vaccinations are for primary prevention, which limits its usefulness to compare with screening mammography, which is secondary prevention. Nonetheless, tetanus vaccination rates remained unchanged from projected during this timeframe.

Our study also has several limitations. First, like all time-series analyses, our results are based on projected trends, thus the strength of our findings relies on the soundness of the projection. There are also limitations inherent to using administrative data. Algorithms for differentiating screening from diagnostic mammograms have not been validated for a database utilizing commercially-based insurance plans. Nevertheless, our conservative assumption favored a null effect. We were unable to determine the effect of potential policy changes within insurance plans that may have affected screening rates. Our study is also unable to determine whether the decrease and then subsequent increase in screening mammogram rates was motivated by patient or physician's preferences or additional unmeasured variables. Given the nature of administrative claims data, we cannot determine whether the decline occurred in women with the lowest breast cancer risk. Furthermore, our study was based on privately-insured women and may not translate to women with other types of health insurance.

These findings demonstrate that the USPSTF update to breast cancer screening guidelines in November 2009 did have a modest initial impact on screening mammography rates for women ages 40–49, but not women 50–64. Unexpectedly, screening mammography has increased beyond projected rates for both age groups in the two-year period following the update. The observed increase in mammography screening might reflect the effect of the economic recovery, media coverage, pro-screening campaigns, clinician advocacy at the point of care, or women's preferences, other factors or their combination. It certainly cannot reflect an improvement in the evidence base in support of mammography in this age group or a manifestation of informed patient preferences. There is need for further investigation not only on delineating benefits and risks of screening mammography for women ages 40–49, but also for more research on how to translate this complex body of evidence into practice, to enable women and their clinicians to engage in effective shared decision making. The events following the USPSTF release also indicate the need for this organization to engage powerful players in ensuring that the signal of their recommendations does not get loss in the strident noise of advocates and pundits. While some may be pleased to see evidence of an increase in mammography screening even among women in their 40s, their enthusiasm should be tempered as we do not know the value of such an increase: we do not know to what extent, if any, the increase in screening mammography rates will result in a detectable decrease in breast cancer morbidity and mortality.

## Supporting Information

Figure S1 **Screening Pap Smear Rates in Women Ages 40–49 and 50–64.**
(TIF)

Text S1 **Statistical Appendix.**
(DOCX)

Text S2 **Sensitivity Analyses.**
(DOCX)
