# Supplementary figures and images for: Impact of the 2009 US Preventive Services Task Force Guidelines on Screening Mammography Rates on Women in Their 40s
Source: PLoS One. 2014 Mar 11;9(3):e91399. doi: 10.1371/journal.pone.0091399 (PMC3950187; doi:10.1371/journal.pone.0091399)

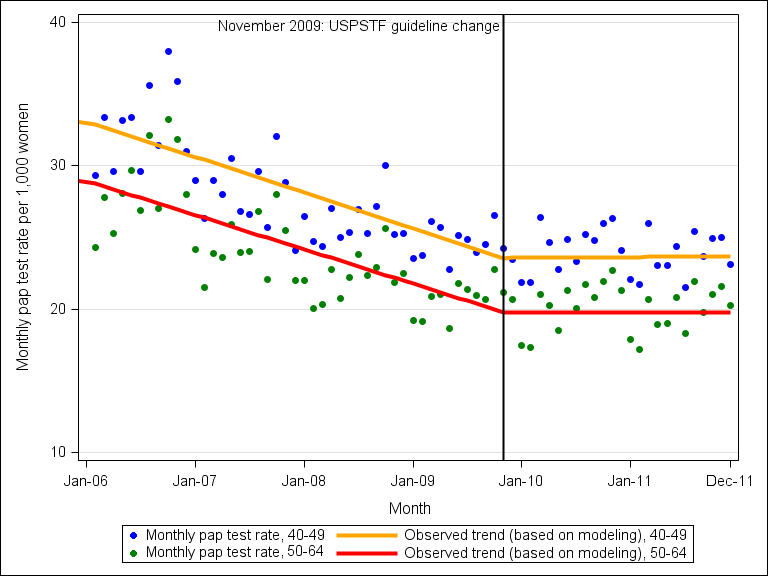

Supplement: Figure S1 — Screening Pap Smear Rates in Women Ages 40–49 and 50–64. (TIF) [file pone.0091399.s001.tif]
