# Supplementary material for: Impact of the 2009 US Preventive Services Task Force Guidelines on Screening Mammography Rates on Women in Their 40s
Source: PLoS One. 2014 Mar 11;9(3):e91399. doi: 10.1371/journal.pone.0091399 (PMC3950187; doi:10.1371/journal.pone.0091399)
Supplement: Text S1 — Statistical Appendix. (DOCX) [file pone.0091399.s002.docx]

**Text S1. Statistical Appendix**

*Details of time series analysis*

To analyze the time series data, we built segmented linear regression models. The time period was divided into two segments: a baseline (January 2006 to October 2009) and a post-USPSTF update period (November 2009 to December 2011).

Specification of the linear regression model

$Y=\beta_{0}+\beta_{1}Time+\beta_{2}Intervention+\beta_{3}Time\_after\_Inv+\varepsilon$

Explanation of terms:

*Y*: Screening mammography rate per 1000 women

*Time*: Time variable to measure trend throughout the observed period

*Intervention*: Indicator variable for the USPSTF update (1 vs. 0)

*Time_after_intervention*: Time variable measuring trend after USPSTF update

*ε*: Error term.

The autocorrelation of the monthly time series data is treated by using autoregressive error models of order 12 using SAS procedure AUTOREG. To diagnose autocorrelation, we used generalized Durbin-Watson statistics and their marginal probabilities. Our analysis showed that such a model is ideal for capturing the autocorrelation in the data, reflecting the fact most mammography screening is done at a yearly interval. Our model also reports high R-square value (0.78 for 40-49 age group, 0.82 for 50-64 age group) indicating good model fitting.
