# Supplementary material for: Impact of the 2009 US Preventive Services Task Force Guidelines on Screening Mammography Rates on Women in Their 40s
Source: PLoS One. 2014 Mar 11;9(3):e91399. doi: 10.1371/journal.pone.0091399 (PMC3950187; doi:10.1371/journal.pone.0091399)
Supplement: Text S2 — Sensitivity Analyses. (DOCX) [file pone.0091399.s003.docx]

**Text S2. Sensitivity analyses**

To check the validity and sensitivity of our modeling approach we considered a few different scenarios by changing the number and cut-off dates of the time segments. Studies have shown that there is often a brief transition period of two to three months, an intervention lag, before changes are made. This may be particularly true in the case of a guideline change as compared with a policy change, the latter which may have a more immediate impact. Furthermore, the initial release of the updated USPSTF breast cancer screening guidelines in November 2009 created a public backlash which prompted the USPSTF to reword the recommendations in an update issued in December 2009, which may have further caused a lag in the intervention effect. Thus, to account for possibly delayed effects of the guideline change, we also defined a separate intervention period from November 2009 to February 2010 in order to allow any change in screening mammography rates in the period immediately after the USPSTF update to be modeled closely. Both an intervention and a trend parameter were assigned to the 4 month period. For the period after February 2010, only a trend parameter was assigned since no sudden level shift is assumed when the intervention period ends. For the 40-49 age group, this model with an autoregressive error of order 12 showed a lower R-square (0.48). The trend parameter is significant (p= 0.02) and has a negative coefficient (-1.18), the intervention parameter is no longer significant at alpha 0.05 level (p= 0.83), the trend parameter for the post-intervention period is positive (0.21) and not significant (p= 0.07). This model does not fit the data better than the model employed in the main analysis, which ended in January 2010. Next, we removed the trend parameter from the intervention period, but the intervention parameter remained insignificant (p=0.28). Then, we redefined the intervention period as ending in January 2010 instead; either way the results are similar. These analyses reveal that there is not convincing evidence to assume a run-in period for the USPSTF update.

We also considered the possible effect of the recent economic recession which is generally considered by economists to be from December 2007 to June 2009. However, the effect of the recession is not easy to detect as it can be confounded with the effect of USPSTF update since its end date is close to the USPSTF update and both may have a negative impact on the mammography screening rates. To account for potential confounding, we fitted a segmented linear regression model to detect the recession effect on the time series data ending in October 2009 before the USPSTF update. A trend parameter is assigned for the recession period which was not found to be significant (p-value 0.25).
